# YTHDC1 Promotes Stemness Maintenance and Malignant Progression in Head and Neck Squamous Cell Carcinoma

**DOI:** 10.1155/2022/7494354

**Published:** 2022-11-12

**Authors:** Junquan Weng, Haidong Fan, Huijuan Liu, Su Tang, Yuyan Zheng

**Affiliations:** Department of Stomatology, Shenzhen People's Hospital (The Second Clinical Medical College, Jinan University, The First Affiliated Hospital, Southern University of Science and Technology), Shenzhen, 518020 Guangdong, China

## Abstract

**Background:**

YTH domain containing 1 (YTHDC1), an N6-methyladenosine (m6A) modification reading protein, plays a key role in regulating RNA translation and degradation. However, the role of YTHDC1 in head and neck squamous cell carcinoma (HNSCC) cancer stem cells remains largely unknown. This study is aimed at investigating the role of YTHDC1 in HNSCC and exploring its role in regulating cancer stem cells.

**Methods:**

RNA sequencing was used to detect differentially expressed genes (DEGs) between SCC9 spheres and SCC9 cells and to uncover molecular pathways and target molecules associated with CSCs. We detected YTHDC1 expression in The Cancer Genome Atlas (TCGA) database data and clinical samples. Subsequently, YTHDC1 gene suppression assays were performed in HNSCC cell lines to investigate the effect of YTHDC1 on tumor cell stemness maintenance, proliferation, and migration capacity. To further confirm the role of YTHDC1 in regulating cancer stem cells in HNSCC, we analyzed online HNSCC single-cell transcriptomic data to investigate YTHDC1 expression patterns at the single-cell level and the correlation of these levels with the expression of stem cell markers.

**Results:**

YTHDC1 expression levels were significantly upregulated in SCC9 spheres, and YTHDC1 was aberrantly expressed in HNSCC tumor tissues. The increased YTHDC1 expression was closely correlated with the clinical characteristics of HNSCC patients. YTHDC1 regulates the malignant phenotype of HNSCC in both *in vivo* and *in vitro* studies. Further single-cell transcriptomic data analysis revealed that YTHDC1 positively correlated with malignant epithelial cell stemness capacity at the single-cell level, and that YTHDC1 was involved in regulating stemness maintenance in HNSCC.

**Conclusions:**

These findings suggest that YTHDC1 may serve as a biomarker for stem maintenance and malignant progression in HNSCC, providing new insights into the treatment of cancer.

## 1. Introduction

Head and neck squamous cell carcinoma (HNSCC) is a group of heterogeneous malignancies of the upper aerodigestive tract, salivary glands, and thyroid gland [1]. Each year, 830,000 people worldwide are diagnosed with head and neck malignancies, and 430,000 die as a result of the disease [2]. Head and neck squamous cell carcinoma accounts for 90% of all head and neck tumors, and these tumors are found in a variety of locations. Approximately 75% of HNSCC cases are associated with tobacco and alcohol usage, whereas a small percentage are caused by human papillomavirus (HPV) infection [3, 4]. The local control rate and quality of life of HNSCC patients have improved due to the development and advancement of surgery and comprehensive treatment, but the overall survival rate has not improved significantly in recent decades, and approximately half of patients die within 1 to 5 years. Thus, the 5-year survival rate remains low primarily due to tumor recurrence or metastasis [1]. Recurrent or metastatic HNSCC is often not amenable to surgical treatment and poorly titrated. Therefore, there is a pressing need to discover novel and effective treatments.

The mechanisms of drug tolerance and metastasis progression must be clarified to establish more effective treatment options. Mounting data indicate that cancer stem cells (CSCs), which are also known as cancer initiating cells, are correlated with cancer initiation [5, 6], progression [6], metastasis [7], resistance to cancer therapy, and recurrence [8, 9]. The stimulation of developmental signaling pathways is the key mechanism through which stem cells develop these malignant characteristics. Classic stem cell signaling pathways, including Oct4, Sox2, Wnt, and Notch, are frequently abnormally elevated in cancer cells [10], allowing tumors to self-renew and proliferate *in vivo*. Functionally, stem cell activation signals could be enriched in cancer stem cells (CSCs), which are linked to resistance to traditional treatment induction of epithelial-mesenchymal transition (EMT). For example, tumor stem cells can upregulate CD47 (“do not eat me”) and PD-L1 (“do not kill me”) signaling, limiting T-cell and macrophage infiltration inside the tumor *in vivo* [11]. CD103-positive tumor stem cell exosomes induce tumor EMT and tumor metastasis in renal clear cell carcinoma [12]. Thus, stem cell signaling is a crucial target for therapy because it drives the core malignant hallmarks of disease development and recurrence.

As a key nuclear m6A reader, YTHDC1 is primarily located in the nucleus and is required for pre-mRNA splicing [13], RNA export [14], and mRNA destabilization [15]. YTHDC1 has recently been discovered to have an important function in stem cells and cancer progression. Chen et al. revealed that YTHDC1 regulates mouse embryonic stem cell self-renewal by recognizing m6A-modified LINE1 RNA [16]. Furthermore, YTHDC1 binds to METTL3 and facilitates METTL3 binding to chromatin in embryonic stem cells, maintaining heterochromatin integrity during mammalian development [17]. Of note, in non-small-cell lung cancer, YTHDC1 increases mRNA stability and mediates immune escape from CD8^+^ T-cell-mediated killing by downregulating PD-L1 ubiquitination and subsequent proteasomal degradation [18]. YTHDC1 facilitates the cytoplasmic export of m6A-modified circHPS5 and downregulates the HMGA2 expression, thereby accelerating hepatocellular carcinoma tumorigenesis [19]. Another study found that YTHDC1 promotes m6A-modified circNSUN2 cytoplasmic export and stabilizes HMGA2 mRNA to promote liver metastasis progression in colorectal cancer [20]. However, the role of HNSCC tumor stem cells remains largely unknown.

In this study, we first compared the transcriptome expression changes between SCC9-derived cancer stem cell-like cells (SCC9 spheres) and the SCC9 cell line using RNA sequencing technology. The YTHDC1 expression was abnormally elevated in SCC9 spheres according to the results. Subsequent data from TCGA database and patient samples indicated that YTHDC1 was significantly elevated in HNSCC tumors and correlated with clinical features. Specific reduction of the YTHDC1 expression in HNSCC cell lines significantly inhibited cell stemness, migration, and proliferative capacity. Further single-cell transcriptomic analysis confirmed that YTHDC1 promoted HNSCC development through regulation of CSCs. Altogether, this study reveals an undetermined role for YTHDC1 in HNSCC, suggesting its potential use as a prognostic marker and therapeutic target for this cancer.

## 2. Materials and Methods

### 2.1. Clinical Sample Acquisition

All specimens (*n* = 6) in this experiment were obtained from HNSCC patients who underwent surgery at Shenzhen People's Hospital. Normal tissues were collected from the tumor margins of the patients. Each patient provided inform consent for tissue analysis prior to surgery, and the study was approved by the Institutional Ethics Committee of Shenzhen People's Hospital.

### 2.2. Cell Lines

Human oral keratinocytes (HOKs) were obtained from ScienCell (USA). The HNSCC cell lines (SCC1, SCC9, SCC15, HN4, HN6) used in this study were purchased from the American Type Culture Collection (ATCC). HOK was maintained in Dulbecco's Modified Eagle Medium/F-12 (DMEM/F12, Gibco) with 10% fetal bovine serum (FBS). HNSCC cell lines were cultured in Dulbecco's Modified Eagle's Medium (DMEM, Gibco) supplemented with 10% FBS and 1% penicillin and streptomycin in a humidified incubator at 37°C and 5% CO_2_.

### 2.3. Cell Transfection

The YTHDC1 gene expression was inhibited using two siRNA sequences as follows: YTHDC1-si-1: GTCGACCAGAAGATTATGATA and YTHDC1-si-2: ATCGAGTATGCAAATATTGAA. To establish YTHDC1 knockdown cell lines, SCC9 and HN6 cells were transfected with siRNA using Lipofectamine 2000 (Thermo Fisher, 11668019). In brief, the siRNA solution was added directly to the dilute transfection reagent using a pipette. The solution was mixed gently by pipetting the up and down. Then, the mixture was incubated for 30 minutes at room temperature. Then, the mixture was overlaid onto HNSCC cells, and the cells were incubated for 6 hours. The cells were assayed 48 hours after the addition of fresh medium.

### 2.4. Immunoblotting

Tissue or cell samples were lysed in RIPA lysis buffer, and the supernatant was collected by centrifugation. The protein lysate was boiled after adding the loading buffer. Protein samples were then separated by 10% sodium dodecyl sulfate-polyacrylamide (SDS–PAGE) gels and subsequently transferred to PVDF membranes. The membranes were blocked with 5% BSA. Primary antibodies were incubated overnight followed by a one-hour incubation with HRP-conjugated secondary antibodies. Protein detection was achieved using the ECL L detection system. The following antibodies were used in the experiment: GAPDH (Proteintech, 10494-1-AP), YTHDC1 (Cell Signaling Technology, 54737S), BMI1 (Abcam, ab269678), SOX2 (Abcam, ab97959), OCT4 (Abcam, ab18976), and NANOG (Abcam, ab109250).

### 2.5. RNA Extraction and Quantitative Real-Time PCR (qRT–PCR)

Total RNA was extracted from HNSCC cells using TRIzol Reagent (Invitrogen, USA) in accordance with the manufacturer's instructions. Reverse transcription was performed using 2 *μ*g of RNA, and qPCR for synthesized cDNA was performed via the HiScript III RT SuperMix for qPCR Kit (Vazyme, China). GAPDH was employed as an internal standard control. The primers used in qPCR were as follows: GAPDH (forward) 5′-AGAAGGCTGGGGCTCATTTG-3′ and GAPDH (reverse), 5′-AGGGGCCATCCACAGTCTTC-3′; YTHDC1 (forward), 5′-AACTGGTTTCTAAGCCACTGAGC-3′ and YTHDC1 (reverse), 5′-GGAGGCACTACTTGATAGACGA-3′.

### 2.6. Cell Proliferation Assay

To determine the effect of YTHDC1 knockdown on cellular proliferation, the transfected cells were seeded into 96-well plates at a density of 1000 cells per well. Cell viability was assessed using the Cell Counting Kit-8 (CCK-8) system (Dojindo, Japan) in accordance with the manufacturer's recommendations at 24, 48, 72, and 96 hours after seeding. In brief, 10 *μ*l of CCK-8 solution was added to each well, and the cells were incubated for 2 h at 37°C in the dark. Subsequently, absorbance was measured at 450 nm.

### 2.7. Cell Migration Assay

Cell migration assays were performed according to the manufacturer's instructions (Falcon, Transwell). Briefly, following transfection with or without si-YTHDC1, cells were synchronized in growth media supplemented with 1% FBS, and migration was induced the next day in the upper chamber of the transwell after seeding into the bottom chamber with medium containing 20% serum. Cells remaining on top of the membrane were scraped with a cotton swab 24 hours later. Cells that migrated into the lower chamber were then fixed with 4% paraformaldehyde and stained with 0.5% crystal violet. Migrated cells were photographed under an inverted microscope and quantified with ImageJ.

### 2.8. Colony Formation Assay

HNSCC cells with or without YTHDC1 deletion (500 cells per well) were transplanted into 6-well plates and cultivated for 1-2 weeks until distinct cell colonies formed. Cell colonies were fixed with 4% formaldehyde and dyed with 0.1% crystal violet after being washed with PBS. The colony-forming capacity of HNSCC cells was measured by counting cell colonies.

### 2.9. Immunohistochemistry

YTHDC1 protein levels in HNSCC tissues were confirmed by IHC. Tumor and normal tissues were formalin-fixed and paraffin-embedded using standard protocols. The 5 mm-thick sections were incubated at 65°C for approximately 1 h followed by a routine dewaxing and rehydration step. Endogenous enzymes were removed with 3% hydrogen peroxide for 10 min and blocked with 5% BSA for half an hour. Then, the samples were incubated overnight with YTHDC1 primary antibody at 4°C. After washing, sections were incubated with biotin-conjugated secondary antibody at RT for 30 min. Then, the signal was amplified for 30 min, developed with DAB developing reagent, and photographed under a microscope. The immunohistochemistry score was calculated by multiplying the staining intensity by the frequency. Staining intensity was defined as 0 (negative), 1+ (weakly positive), 2+ (positive), or 3+ (strongly positive). Frequency was defined as the percentage of positive cells.

### 2.10. Sphere Formation Assay

Stem cell medium containing 5000 SCC9 or HN4 cells/ml was placed in 6-well ultralow plates according to a previously described method [21]. Then, the cells were cultured in an incubator at 37°C with 5% CO_2_ for 1-2 weeks. Every two days, the stem cell media was refreshed. Photographs and counts were collected under a microscope after sphere formation. We obtained a group of cells with greater spheroid formation ability termed SCC9 spheres after five generations of sphere formation culture.

### 2.11. RNA Sequencing

Total RNA was extracted from two biological replicates of the SCC9 cell line and SCC9 spheres for RNA-sequencing profiling. The Illumina TruSeq Stranded Total RNA Library Prep Kit (RS-122-2201) was used to construct the sequencing libraries. The libraries were sequenced in paired-end mode on one lane of the Illumina HiSeq2500 instrument. Then, *R* software was used to analyze differentially expressed genes. By default, a gene was considered significantly differentially expressed when the *p* value was ≤0.05, and the fold change was greater than or equal to 1.5. The RNA sequencing datasets in this study were deposited on GEO (GSE205380, https://www.ncbi.nlm.nih.gov/geo/query/acc.cgi?acc=GSE205380).

### 2.12. Gene Enrichment Analyses

GSEA software downloaded from the GSEA website (https://www.gsea-msigdb.org/gsea/downloads.jsp) was used to determine signaling pathways that are linked between the SCC9 sphere group and the SCC9 cell line group. Hallmark gene sets from the Molecular Signature Database (MSigDB) were used in GSEA version 4.0 with 1,000 permutations. The major statistic for GSEA was the normalized enrichment score (NES). Significantly enriched gene sets were determined by FDR < 0.25, *p* < 0.05, and NES > 1.0.

### 2.13. Animal Studies

For the *in vivo* subcutaneous transplanted model studies, four-week-old BALB/C female nude mice were randomly divided into two groups (*n* = 6 per group). HNSCC cell lines transfected with YTHDC1-deficient or control plasmid were subcutaneously injected into mice to explore the effect *in vivo*. After two weeks, all the mice were sacrificed, and intact xenograft tumors were obtained. The volume of transplanted tumor volume was determined as follows: length × width^2^/2.

### 2.14. Single-Cell Analysis

In this study, scRNA-seq data of human head and neck squamous carcinomas published in 2017 were used for analysis. The scRNA-seq data of a total of 5902 cells were obtained from the Gene Expression Omnibus (GEO, http://www.ncbi.nlm.nih.gov/geo/) database (GEO number GSE103322), and 2215 malignant cell data were chosen for further analysis. The Seurat *R* package was used to analyze the data. To explore the relationship between YTHDC1 and the pathogenesis of HNSCC, the upregulated DEGs obtained from single-cell analysis were subjected to KEGG and Gene Ontology analysis using DAVID Bioinformatics Resources 6.8 (https://david.ncifcrf.gov).

### 2.15. Statistical Analysis

Experimental data are presented as the mean ± standard error of the mean (SEM) or standard deviation (SD). Two-tailed Student's *t*-test was used to determine the significance of significant differences between the two groups. The relationship between the specific genes in TCGA database and the single-cell transcriptomic analysis data was assessed using the Pearson correlation test. Significance levels are ^∗^*p* < 0.05, ^∗∗^*p* < 0.01, ^∗∗∗^*p* < 0.001, and ^∗∗∗∗^*p* < 0.0001.

## 3. Results

### 3.1. SCC9 Spheres Exhibit Inflated CSC-Like Properties

**Figure 1:**
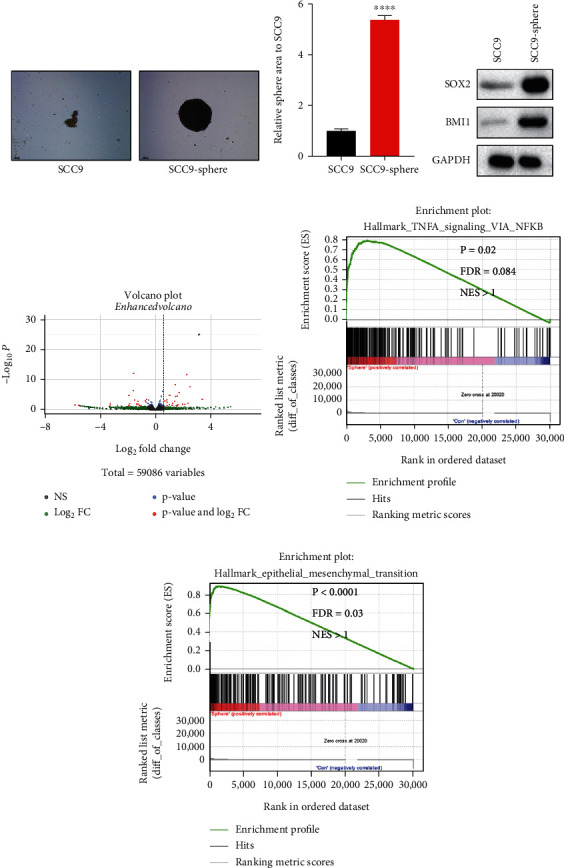
SCC9-sphere exhibits inflated CSC-like properties. (a) The comparison of sphere formation ability between SCC9-sphere and SCC9 cell line. Scale bar, 100 *μ*m. (b) Bar graph showed SCC9-sphere enhanced the sphere formation ability compare with the SCC9 cell line. (c) SOX2 and BMI1 protein levels in two groups. (d) Volcano plot of differentially expressed genes from RNA sequencing results. (e, f) GSEA comparing the SCC9-sphere group with the SCC9 cell line group in TNFA and EMT signaling pathway. Nominal *p* values, false discovery rate (FDR), and normalized enrichment score (NES) were shown.

We cultured cell sphere formation for five consecutive generations to obtain cells with enhanced stemness and compared the sphere formation efficiency of the fifth-generation sphere with that of the original SCC9 cell line. SCC9 spheres exhibited greater sphere formation efficiency and stemness (Figures 1(a) and 1(b)). Subsequent western blotting analysis, which revealed higher protein expression levels of the stemness-related markers BMI1 and SOX2 in SCC9 spheres (Figure 1(c)), yielded similar results.

To investigate the molecular mechanisms by which tumor stem cells regulate HNSCC progression, we performed RNA sequencing of the SCC9 cell line and SCC9 spheres. The SCC9 cell line was employed as a control group, and the differentially expressed genes (DEGs) were classified into upregulated, nonsignificant (nonsig.), and downregulated groups and illustrated by volcano plots (Figure 1(d)). We identified 23 downregulated genes and 23 upregulated genes. Among the highly expressed genes, YTHDC1 was identified as a downstream molecule given that it demonstrated the highest upregulation and highly significant differences (Figure 1(d)). Furthermore, Gene Set Enrichment Analysis (GSEA) revealed that several representative oncogenic signaling pathways, including tumor necrosis factor (TNF) signaling via NF-kappaB and epithelial mesenchymal transition signaling, were enriched in SCC9 spheres (Figures 1(e) and 1(f)), indicating that CSCs drive tumorigenesis, invasion, and chemotherapy tolerance in HNSCC.

### 3.2. YTHDC1 Is Aberrantly Expressed in HNSCC and Associated with Cancer Cell Stemness

**Figure 2:**
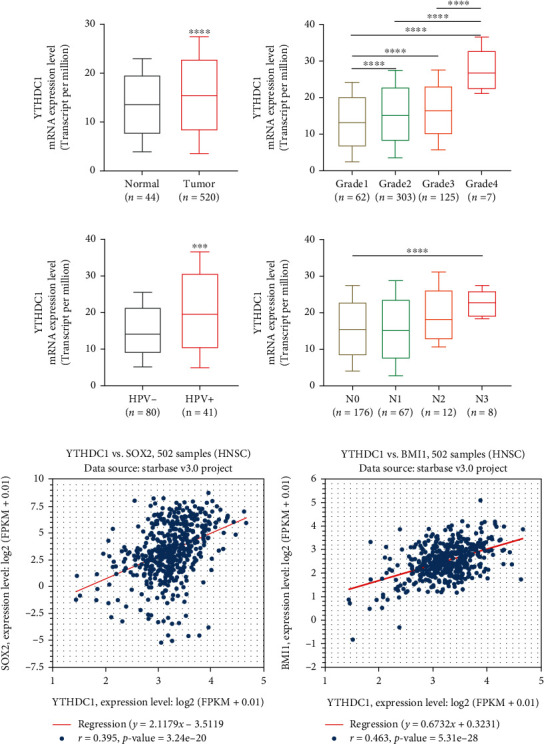
YTHDC1 aberrantly expressed in HNSCC and was associated with cancer cell stemness. (a) The relative mRNA expression of YTHDC1 in tumor and normal tissues in HNSCC in TCGA database. The comparison of the YTHDC1 mRNA expression in (b) individual tumor grades, (c) HPV infection status, and (d) different nodal metastasis status. (e) The Pearson correlation between YTHDC1 and SOX2 mRNA expression in HNSCC according to TCGA data. (f) The Pearson correlation between YTHDC1 and BMI1 mRNA expression in HNSCC according to the TCGA data.

To investigate the role of YTHDC1 in HNSCC, we first analyzed its expression pattern. YTHDC1 mRNA was abnormally upregulated in HNSCC tumor tissues compared to normal tissues according to TCGA data (Figure 2(a)). Specifically, the increased YTHDC1 expression was associated with the following clinical characteristics of HNSCC patients: higher tumor grade (Figure 2(b)), positive HPV presentation (Figure 2(c)), and increased lymph node metastasis (Figure 2(d)). Subsequently, we also analyzed the coexpression of YTHDC1 with stemness-related markers in the StarBase database and found that the YTHDC1 mRNA expression was significantly and positively correlated with the SOX2 and BMI1 expression in 502 HNSCC samples (Figures 2(e) and 2(f)). This finding also indicates that YTHDC1 plays a role in regulating CSCs in HNSCC.

### 3.3. YTHDC1 Was Overexpressed in HNSCC Patients

**Figure 3:**
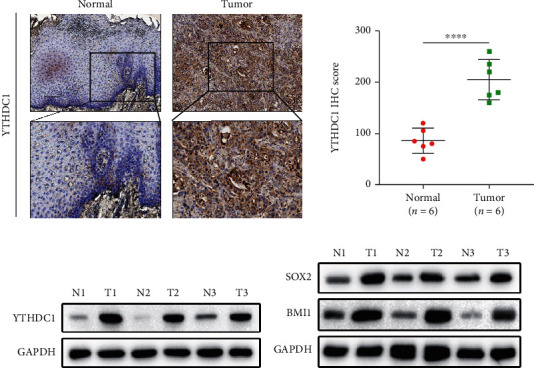
YTHDC1 was overexpressed in HNSCC patients. (a) Representative graph of YTHDC1 staining in tumor and normal tissues of HNSCC patients (left) and the IHC score quantification (right) (*n* = 6). Scale bar, 100 *μ*m. (b) The YTHDC1 expression in patients HNSCC tissues and normal tissues detected by immunoblotting analysis (*n* = 3). (c) The SOX2 and BMI1 protein expression in patient HNSCC tissues and normal tissues detected by immunoblotting analysis (*n* = 3).

To further assess YTHDC1 expression levels in HNSCC patients, we performed immunostaining to localize and identify YTHDC1 protein molecules *in vivo*. The findings revealed that the higher YTHDC1 protein expression was observed in HNSCC patient samples compared with paired normal tissues (*n* = 6) (Figure 3(a)). Subsequent western blotting assays of patient protein specimens supported this conclusion (Figure 3(b)). In addition, our data revealed that SOX2 and BMI1 expression levels in tumor samples were also higher than those in normal tissues (Figure 3(c)). These results suggest that YTHDC1 and stemness-related markers are aberrantly overexpressed in HNSCC, and that YTHDC1 plays an important procancer role.

### 3.4. Inhibition of YTHDC1 Expression Significantly Limits Tumor Cell Stemness Maintenance, Migration, and Proliferation

**Figure 4:**
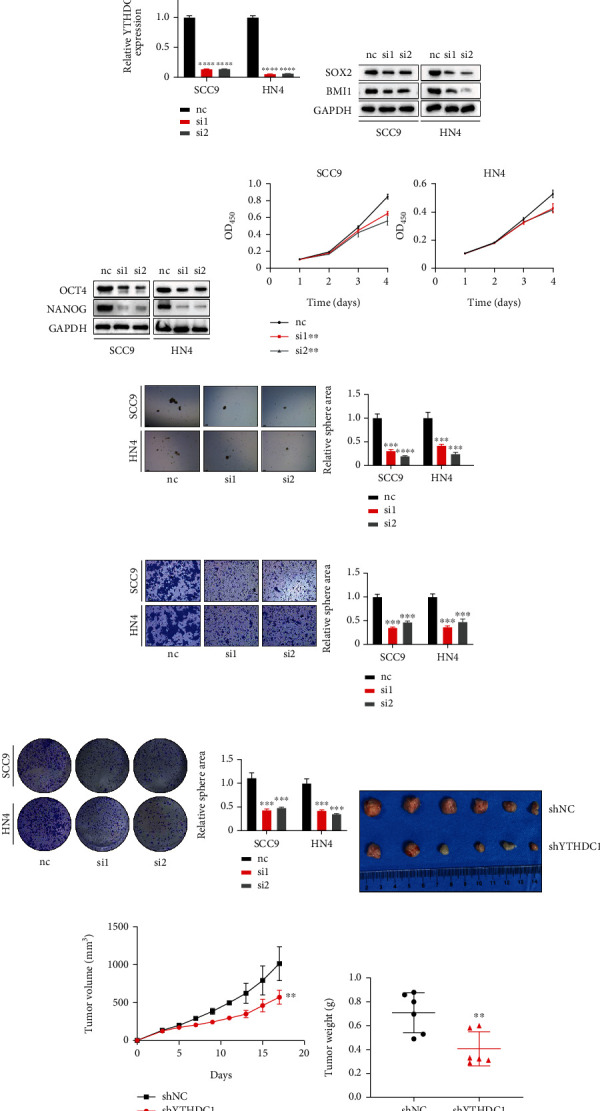
Inhibition of the YTHDC1 expression significantly limits tumor cell stemness maintenance, migration, and proliferation. (a) Western blotting of YTHDC1 in six HNSCC cell lines. (b) Western blotting of YTHDC1 in SCC9 and HN4 with or without YTHDC1 knockdown. (c) qPCR data of YTHDC1 in SCC9 and HN4 with or without YTHDC1 knockdown. (d) SOX2 and BMI1 protein expression in SCC9 and HN4 with or without YTHDC1 knockdown. (e) The OCT4 and NANOG protein expression in SCC9 and HN4 with or without YTHDC1 knockdown. (f). SCC9 and HN4 cell proliferation ability was suppressed after the YTHDC1 expression was decreased. (g) Sphere formation ability of SCC9 and HN4 cell lines was inhibited after the YTHDC1 expression was suppressed. (h) Representative images and quantification of migration assay of SCC9 and HN4 cell lines with or without YTHDC1 knockdown. (i) Representative images and quantification of colony formation assay of SCC9 and HN4 cell lines with or without YTHDC1 knockdown. (j). Tumor xenograft experiment in nude mice detects the effect of YTHDC1 on tumor growth in vivo (*n* = 6 in each group). (k) Tumor growth curve. The quantitative value of tumor volumes is plotted, and tumor volumes are measured once two days after injected. (l) Scatter diagram shows tumor weight.

To further clarify the function of YTHDC1 *in vitro*, we compared the protein expression levels of YTHDC1 in five HNSCC cell lines and one normal cell line and found that its expression was higher in SCC9 and HN4 cells (Figure 4(a)). Therefore, we selected the two cell lines for the subsequent functional study. First, we constructed YTHDC1 knockdown cells using siRNA targeting YTHDC1 and confirmed YTHDC1 knockdown with western blotting and qPCR (Figures 4(b) and 4(c)). Interestingly, inhibition of the YTHDC1 expression in the SCC9 and HN4 cell lines resulted in a reduction in the expression of SOX2, BMI1, OCT4, and NANOG (Figures 4(d) and 4(e)), indicating a significant decrease in the stemness of HNSCC cells.

Based on these results, we then investigated whether YTHDC1 could regulate CSC properties in HNSCC cell lines. The data revealed that the cell proliferation and sphere formation ability of cells with the reduced YTHDC1 expression was severely hindered (Figures 4(f) and 4(g)). In addition, cell migration assays and cell clone formation assays showed that YTHDC1 knockdown also significantly suppressed HNSCC cell migration and proliferation (Figures 4(h) and 4(i)). Consistent with the *in vitro* study results, silencing YTHDC1 expression in SCC9 cells dramatically retarded tumor growth compared with the control cells in nude mice (Figure 4(j)), as confirmed by the measurement of tumor volume and tumor weight (Figures 4(k) and 4(l)). In conclusion, YTHDC1 is required for HNSCC cells to maintain their self-renewal, migration, and proliferation abilities.

### 3.5. Validation of the Role of YTHDC1 in Regulating Stemness Maintenance at the Single-Cell Level

**Figure 5:**
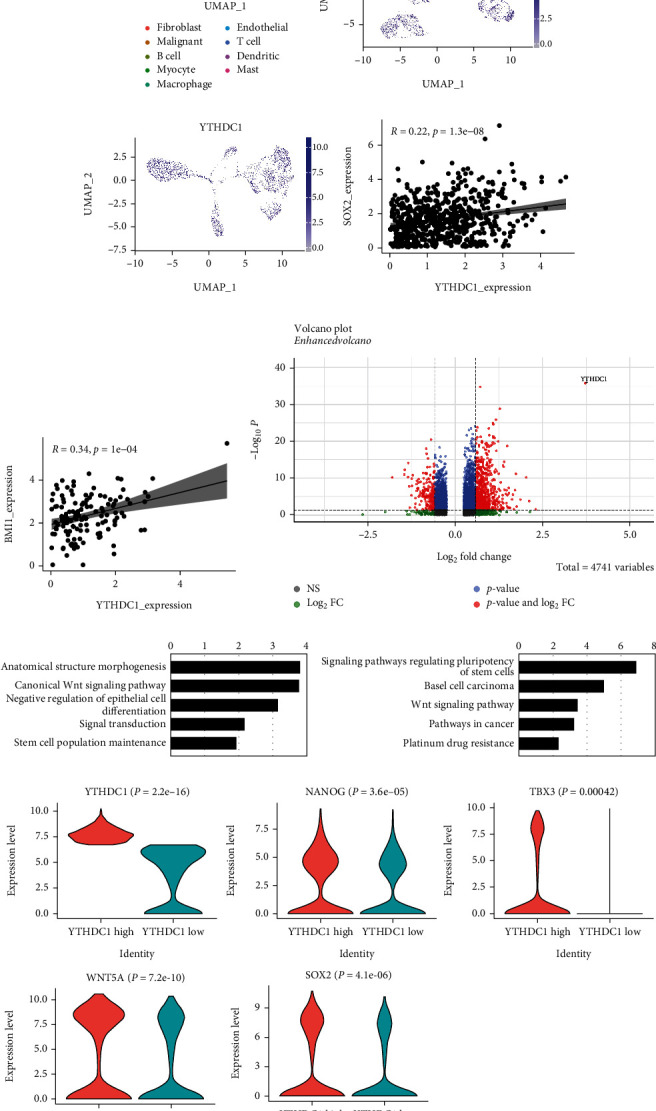
Validation of the role of YTHDC1 in regulating stemness maintenance at the single cell level. (a) The UMAP plot of scRNA-seq cell data labeled by cell type. (b) The UMAP plot of the YTHDC1 expression in all the clusters. (c) The UMAP plot of cancer cells from HNSCC tissues shows the YTHDC1 expression pattern. (d) The Pearson correlation between YTHDC1 and SOX2 mRNA expression according to the single cell RNA data. (e) The Pearson correlation between YTHDC1 and BMI1 mRNA expression according to the single cell RNA data. (f) Volcano plot shows differentially expressed genes between YTHDC1 high and YTHDC1 low cells. (g) GO analysis of the upregulated genes in the YTHDC1 high expression cell population. (h) KEGG analysis of the upregulated genes in the YTHDC1 high expression cell population. (i) Violin plot depicts the expression of stem cell-related markers of YTHDC1 high and YTHDC1 low cells.

To further confirm the regulatory CSC function of YTHDC1 in HNSCC, we analyzed an online single-cell transcriptomic analysis dataset that defines the transcriptional profile of HNSCC single cells (GSE103322). The data partitioned cells into one malignant cluster (malignant cells) and eight nonmalignant cells (fibroblasts, B cells, myocytes, macrophages, endothelial cells, T-cells, dendritic cells, and mast cells) (Figure 5(a)). Further analysis showed the increased YTHDC1 expression in malignant cells compared with nonmalignant cells (Figure 5(b)). Then, we evaluated the YTHDC1 expression in 2,215 of these malignant epithelial cells. YTHDC1 was aberrantly expressed in most malignant epithelial cells (Figure 5(c)). The mRNA expression level of YTHDC1 was significantly and positively correlated with both SOX2 and BMI1, confirming the prior findings (Figures 5(d) and 5(e)). We then divided malignant epithelial cells into YTHDC1 high- and YTHDC1 low-expression cell populations and used volcano plots to show the DEGs. We found 917 differentially expressed genes between the two populations, including 188 downregulated genes and 729 upregulated genes. A total of 729 genes (including WNT2, SOX9, NANOG, TBX3, WNT5A, and SOX2) were significantly increased in the YTHDC1 high group (Figure 5(f)). We then performed enrichment analysis of the upregulated DEGs. Gene Ontology analysis associated with biological process enrichment revealed that these genes were selectively enriched for the canonical Wnt signaling pathway, signal transduction, and stem cell population maintenance, which may be involved in tumor cell messaging and maintenance of tumor stemness (Figure 5(g)). Next, we performed KEGG enrichment analysis of DEGs to systematically examine cellular pathways affected by YTHDC1 and found that signaling pathways for the maintenance of stem cell pluripotency were the most enriched (Figure 5(h)). More precisely, multiple marker genes of stemness-related pathways, such as NANOG, TBX3, WNT5A ,and SOX2, were highly upregulated compared with the YTHDC1 low group (Figure 5(i)). These findings support YTHDC1's critical involvement in controlling HNSCC tumor stemness at the single-cell level.

## 4. Discussion

In recent years, with continuous research in the field of molecular biology, CSCs have been found to play an important role in tumor development, invasion, metastasis, chemoresistance, and radioresistance [22–24]. The repopulation and differentiation of CSCs often lead to disease recurrence and play an important role in poor prognosis. CSCs also provide new targets for treatment. Based on the increasing understanding of tumor stem cell biology, multiple new therapeutic strategies targeting cancer stem cells have been proposed and have become a recent hot topic in oncology treatment. For example, scientists from the University of California and other institutions have developed a novel DNA therapy that may be expected to help remove cancer stem cells from mice and help treat multiple myeloma [25]. Another study successfully identified kidney cancer stem cells and found that blocking Wnt and Notch has the potential to treat kidney cancer [26]. The tumor cell sphere formation assay is the gold standard method for determining the stemness of tumor cells. In our study, we obtained aberrantly highly expressed genes in HNSCC tumor stem cells by comparing SCC9 sphere and the SCC9 cell line and showed potential therapeutic targeting ability given the close correlation of these genes with clinical features.

Epigenetic modification is a form of gene expression regulation that affects gene transcription and translation without altering nucleotide sequences. These modifications regulate and subsequently affect gene expression at the DNA level as well as chromatin structural modifications, RNA stability, and transcriptional activity, which mainly includes DNA methylation modification, histone covalent modification, chromatin remodeling, and RNA interference [27, 28]. Currently, epigenomics based on N6-methyladenosine (m^6^A) modifications has become a hot research topic.

Recent advances have highlighted that m^6^A RNA modification plays an important role in cancer biology and CSCs [29–31]. Recent advances in m6A readers have highlighted that m^6^A RNA modification plays an important role in cancer biology and CSCs. For instance, YTHDF2, an m^6^A reader protein, promotes the proliferation of liver cancer tumor stem cells and promotes tumor metastasis [32]. In another study, researchers found that YTHDF1 promotes tumor development by maintaining the stemness of tumor cells in colorectal cancer [33].

In our study, we described in detail the regulatory mechanism of YTHDC1 in HNSCC tumor stem cells. YTHDC1 was identified by RNA-seq analyses, and its expression was closely correlated with the clinical features of HNSCC. Specifically, previous studies have also characterized the genetic alterations and prognostic value of YTHDC1 in HNSCC. The YTHDC1 expression is abnormally elevated in HNSCC patients and is associated with poor prognosis [34, 35], whereas another analysis showed that patients with high YTHDC1 expression obtained a better prognosis [36]. Therefore, the role of YTHDC1 in HNSCC patients remains controversial. Notably, we found that YTHDC1 mRNA expression levels were significantly and positively correlated with those of BMI1 and SOX2, which are HNSCC tumor stem cell-specific indicators. These data suggest that YTHDC1 is associated with tumor stemness regulation in HNSCC.

The patient sample data also demonstrated that YTHDC1 was abnormally highly expressed in tumors, suggesting its potential procancer role. We knocked down YTHDC1 in two cell lines with high YTHDC1 expression to inhibit cell stemness, migration, and proliferation, further revealing its role in promoting the development of HNSCC. Therefore, *in vitro* experiments demonstrated that YTHDC1 promotes malignant tumor progression in HNSCC and may represent a poor prognostic indicator for patients. Subsequently, we analyzed single-cell transcriptomic analysis data to verify the correlation between YTHDC1 and tumor stemness. The results demonstrated that YTHDC1 was significantly and positively correlated with BMI1 and SOX2 mRNA expression levels in HNSCC tumor cells, and that cells with high YTHDC1 expression coexpressed stemness pathway-related genes. This finding is also consistent with our previous *in vitro* results, further demonstrating the role of YTHDC1 in regulating tumor cell maintenance in stem cell lines and providing ideas for further studying the functional mechanism of YTHDC1 in HNSCC. Taken together, these data support the notion that YTHDC1 promotes HNSCC pathogenesis through manipulation of the CSC stemness pathway.

In conclusion, our findings reveal a role for YTHDC1 in regulating the tumor stem cell pathway in HNSCC. YTHDC1 promotes HNSCC progression through manipulation of the stemness-related pathway. The present study however has significant limitations. A key issue is the absence of experimental data from verified animal models. To confirm the molecular process, further sophisticated experimental techniques are needed. Although further studies are needed in the future to reveal the oncogenic mechanisms of YTHDC1, our current study lays the foundation for better therapeutic strategies.

## Data Availability

The data used to support the findings of this study are included within the article.
